# How do supported employment programs work? Answers from a systematic literature review

**DOI:** 10.1007/s10775-022-09533-3

**Published:** 2022-02-27

**Authors:** Larissa M. Sundermann, Sigrid Haunberger, Fiona Gisler, Zuzanne Kita

**Affiliations:** 1 HSO Wirtschafts- und Informatikschule (HSO Business School), Andreasstrasse 15, 8005 Zurich, Switzerland; 2 School of Social Work, ZHAW Zurich University of Applied Sciences, Pfingstweidstrasse 96, P.O. Box, 8037 Zurich, Switzerland; 3 School of Management & Law, ZHAW Zurich University of Applied Sciences, Theaterstrasse 17, 8401 Winterthur, Switzerland

**Keywords:** Impact model, Realist evaluation, Social work

## Abstract

Many studies have found that supported employment (SE) has effectively helped people with severe mental illness obtain and maintain competitive employment. However, most SE studies have asked “What works?” rather than discerning what works for whom, in what circumstances, in what respects and how. It is important to understand the outcomes of SE and identify the impact factors (contexts and mechanisms) that can trigger them. Four literature databases were searched for studies that analyzed counseling settings. Overall, 104 publications met the inclusion criteria. The review showed that most of the research on SE programs were one-dimensional, looking at either the effects of SE programs, the client or the professional, or the relationship between clients and professionals. The model reveals that impact factors are interconnected and can have a cumulative impact on the client, professionals, and the environment.

**Supplementary Information:**

The online version contains supplementary material available at 10.1007/s10775-022-09533-3.

## Résumé

**Comment fonctionnent les programmes d'emploi assisté? Réponses d'une revue systématique de la littérature**

De nombreuses études ont montré que l'emploi assisté (SE) a efficacement aidé les personnes atteintes de maladie mentale grave à obtenir et à conserver un emploi compétitif. Cependant, la plupart des études SE ont demandé “Qu'est-ce qui fonctionne?” plutôt que de discerner ce qui fonctionne pour qui, dans quelles circonstances, à quels égards et comment. Il est important de comprendre les résultats de l'ES et d'identifier les facteurs d'impact (contextes et mécanismes) qui peuvent les déclencher. Quatre bases de données de la littérature ont été consultées pour des études analysant les contextes de conseil. Au total, 104 publications répondaient aux critères d'inclusion. L'examen a montré que la plupart des recherches sur les programmes SE étaient unidimensionnelles, examinant soit les effets des programmes SE, le client ou le professionnel, soit la relation entre les clients et les professionnels. Le modèle révèle que les facteurs d'impact sont interconnectés et peuvent avoir un impact cumulatif sur le client, les professionnels et l'environnement.

## Abstract (de)

**Wie funktionieren Programme der Unterstützten Beschäftigung? Antworten aus einer systematischen Literaturrecherche**

Viele Studien haben ergeben, dass unterstützte Beschäftigung (SE) Menschen mit schweren psychischen Erkrankungen effektiv dabei geholfen hat, eine wettbewerbsfähige Beschäftigung zu finden und aufrechtzuerhalten. Die meisten SE-Studien haben jedoch gefragt: “Was funktioniert?” anstatt zu erkennen, was für wen, unter welchen Umständen, in welcher Hinsicht und wie funktioniert. Es ist wichtig, die Ergebnisse von SE zu verstehen und die Einflussfaktoren (Kontexte und Mechanismen) zu identifizieren, die sie auslösen können. Es wurden vier Literaturdatenbanken nach Studien durchsucht, die Beratungssettings analysierten. Insgesamt erfüllten 104 Publikationen die Einschlusskriterien. Die Überprüfung zeigte, dass die meisten Untersuchungen zu SE-Programmen eindimensional waren und entweder die Auswirkungen von SE-Programmen, den Klienten oder die Fachkraft oder die Beziehung zwischen Klienten und Fachkräften betrachteten. Das Modell zeigt, dass Einflussfaktoren miteinander verbunden sind und sich kumulativ auf den Klienten, die Fachkräfte und die Umwelt auswirken können.

## Resumen

**¿Cómo funcionan los programas de empleo con apoyo? Respuestas de una Revisión Sistemática de la Literatura**

Muchos estudios han encontrado que el empleo con apoyo (SE) ha ayudado efectivamente a las personas con enfermedades mentales graves a obtener y mantener un empleo competitivo. Sin embargo, la mayoría de los estudios de SE han preguntado "¿Qué funciona?" en lugar de discernir qué funciona para quién, en qué circunstancias, en qué aspectos y cómo. Es importante comprender los resultados de la ES e identificar los factores de impacto (contextos y mecanismos) que pueden desencadenarlos. Se realizaron búsquedas en cuatro bases de datos bibliográficas en busca de estudios que analizaran entornos de asesoramiento. En total, 104 publicaciones cumplieron los criterios de inclusión. La revisión mostró que la mayoría de las investigaciones sobre los programas de SE eran unidimensionales y analizaban los efectos de los programas de SE, el cliente o el profesional, o la relación entre los clientes y los profesionales. El modelo revela que los factores de impacto están interconectados y pueden tener un impacto acumulativo en el cliente, los profesionales y el medio ambiente.

## Introduction

For many people, work means belonging to a society. It is an essential part of their lives and improves their self-esteem, helps establish social relationships, and sets life goals (Knaeps et al., 2012). Unemployment might negatively affect individuals and their families and predict negative societal and economic implications. It can cause increased psychological and physical problems as well as higher mortality and suicide rates (Hedley et al., 2017). Some, especially those with a form of disability or other impairments, are particularly affected, as they often struggle to attain a competitive job. For them, a diverse set of vocational rehabilitation (VR) programs can help them find employment. Most of these programs are categorized as supported employment (SE) programs, which showed their effectiveness in many ways (Drake & Wallach, 2020; Williams et al., 2016). SE is a so-called “place-then-train” model (Corbière et al., 2013; Grigorovich et al., 2017) and is defined as a value-based and person-centered approach to assist and support people with limited access to the labor market in obtaining and retaining paid work in companies in the first labor market. It inverts the traditional VR approach, placing the client in a work environment first and providing support within the work setting rather than training the client before finding a job (Homa & DeLambo, 2015). Some practices, such as the individual placement and support (IPS) model of SE for people with severe mental illness (SMI), are evidence-based and systematic, to help achieve competitive employment (Bond et al., 2012). IPS is based on eight principles: eligibility based on client choice, focus on competitive employment, integration of mental health and employment services, attention to client preferences, work incentives planning, rapid job search, systematic job development, and individualized job supports (Bond et al., 2012). Other programs vary from segregated day programs to competitive employment as well as segregated work settings such as sheltered workshops (Verdugo et al., 2006).

Concerning outcomes, meta-analyses have found that SE, especially high-fidelity IPS, has been effective in helping individuals with SMI attain and maintain competitive employment compared with other VR interventions such as prevocational training, sheltered employment, or psychiatric services only (Hedley et al., 2017; Ng & Shanks, 2020). Program participants holding competitive jobs for a constant period show benefits such as improved self-esteem and better symptom control on the one hand (Hedley et al., 2017). On the other hand, SE enrollment has no systematic effect on nonvocational outcomes, either on undesirable outcomes, such as rehospitalization, or on valued outcomes such as improved quality of life (Bond, 2004). Studies on the cost efficiency of SE conclude that individuals with disabilities fare better financially from working in the community than in sheltered workshops and that SE appears to be more cost effective than sheltered workshops over the entire “employment cycle” and returns a net benefit to taxpayers. Cimera (2000) also points out that individual placements appear to be the most cost-efficient SE methods (Bond et al., 2001; Williams et al., 2016).

Even though different types of programs exist, common features are identifiable. SE is an evidence-based practice with multiple components designed to help adults with mental disorders or cooccurring mental and substance use disorders find and sustain competitive employment (Marshall et al., 2014). Competitive employment is characterized by the following factors: jobs with permanent status, paid minimum wage, and jobs not set aside for people with disabilities (Salyers et al., 2004). SE is usually defined by the following principles: (1) inclusion of all clients who want to work; (2) integration of vocational and clinical services; (3) emphasis on competitive employment; (4) fast job search without prevocational skills training; (5) job development by the employment specialist; (6) attention to the client’s preferences, skills, and experiences about their desired work and disclosure of mental illness to future employers; (7) benefits counseling; and (8) ongoing support services after a job is obtained (Mueser & McGurk, 2014).

Regarding the diverse set of programs, service providers, and target groups, it is not surprising that questions remain about the impact and the comparability of these programs since even though SE programs are mostly specialized for one specific target group, success rates are relatively low (Hedley et al., 2017; Knaeps et al., 2012). There is a need to identify appropriate factors that contribute to the success of these programs. In contrast to traditional systematic literature reviews (SLR) this contribution presents a research synthesis, which is based on the realist approach.^1^ It provides an explanatory analysis of complex social interventions or programs, like SE, aimed at discerning what works for whom, in what circumstances, in what respects and how, which at the same time corresponds to the main question. The results of the review combine theoretical understanding and empirical evidence and focus on explaining the relationship between the context in which the SE program is applied, the mechanisms by which it works and the outcomes which are triggered (the so-called CMO configurations, Pawson & Tilley, 1997; Pawson et al., 2005). These are then presented clearly in an SE impact model.

## Method

Since the number of available scientific publications on SE increases rapidly each year, it is useful to summarize individual studies on a single topic clearly and concisely. An SLR can provide an overview of the state of research. This method is characterized by a systematic approach to the selection, inclusion, and analysis of literature sources (Machi & Mcevoy, 2016; Pawson et al., 2005). A detailed study protocol and evaluation plan are prepared, and the literature search is carried out in a priori defined literature databases as well as according to a priori defined inclusion and exclusion criteria (Ressing et al., 2009). Finally, the results are summarized qualitatively by contexts, mechanisms, and outcomes, which correspond to a realist review (Pawson et al., 2005). The further structure of the contribution follows the six literature review steps proposed by Machi and Mcevoy (2016), which is briefly explained below.

*Step 1 Select a topic* and *Step 2 Develop the tools for argument*: The search inquired which impact factors (i.e., contexts, mechanisms) in a counseling setting influence the effectiveness of SE programs (i.e., outcomes), guaranteeing a broad search approach.

*Step 3 Search the literature*: Four electronic databases were searched: (1) *Web of Science*, (2) *PsycInfo*, (3) *WISO,* and (4) *Social Science Abstracts*. The focus was on post-1999 publications because the debate about impact began during that time, and publications have continued to increase since then. Before deciding on this search term, the most common synonyms of “effectiveness” and “supported employment” were combined, including “impact,” “impact factors,” “effects,” “efficacy,” “vocational rehabilitation,” and “individual placement and support.” This procedure allowed for the inclusion of the largest number of relevant articles that have been excluded from a combination of the other search terms. For example, when evaluating the search terms, “impact” was excluded as the results were less satisfactory than for “effectiveness” and yet the relevant “impact” articles occurred in the results. The chosen search term, therefore, used a combination of “effectiveness” AND “supported employment” (German: “Wirkung” UND “Arbeitsintegration”) to include as many publications as possible.^2^ We set the boundaries for the systematic review by defining inclusion and exclusion criteria which can be seen in Table 1. No studies were excluded based on the evaluation of quality. This inclusive approach ensured that the search captured a range of research methods and varied elements of SE programs. Consistent with the approach of realist review (Pawson et al., 2005), there was no reason to exclude literature sources based on the research design.

**Table 1:** Inclusion and exclusion criteria of the SLR

	Inclusion criteria	Exclusion criteria
Type of publication	Full text (e.g., articles, books, dissertations, etc.)	Study comments, introductions to special issues, conference abstracts
Language	German, English	All other
Date of publication	2000–2018	All other
Geographic context	All	–
Research field	All	–
Research group	Adults, individuals ready to work	Underage individuals, children
Research focus	1. SE programs, impact factors/impact/effectiveness 2. Clear connection between impact and SE programs	1. No clear focus on SE programs 2. SE integrated into another program 3. Programs not containing professional settings (e.g., support groups, self-help groups, software programs, or peer counseling) 4. Economic interest 5. Effects of reintegration into labor market 6. Political decisions 7. Analyzing SE programs with no interest in impact factors, impact, or effectiveness

**Figure 1:**
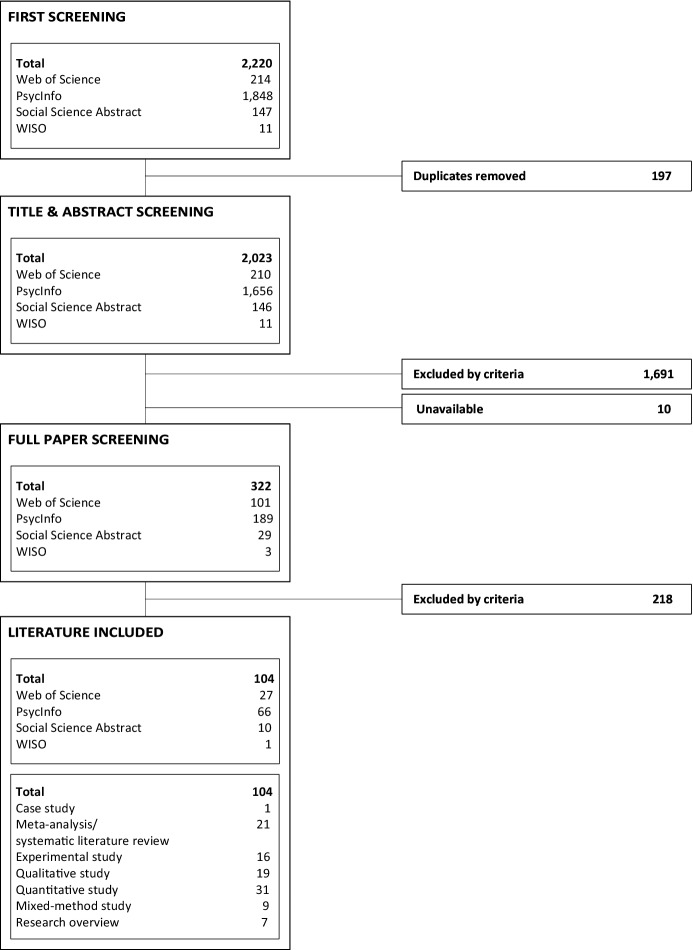
Flowchart of the literature search results and inclusion/exclusion criteria

*Step 4 Survey the literature* A multistep screening process was executed to identify relevant publications. First, a screening of all 2023 titles eliminated clearly irrelevant articles that did not meet the inclusion criteria. Of the remaining ones, abstracts were checked for relevance. Because of this screening, 1691 publications did not meet the inclusion criteria and were not relevant to the study context. Afterward, full-text availability was proven, excluding 10 unavailable publications. The remaining 322 publications were evaluated by two authors for content based on their full text. After finding no discrepancies, the first author conducted a full review of the identified articles. Only 104 publications that met all the inclusion criteria were analyzed and coded. Those that did not meet the inclusion criteria were flagged for removal. Afterward, two reviewers evaluated those studies. The decision whether the study should be removed or included was based on consensus. Figure 1 provides an overview of the search results at each stage of the process. Of the 104 identified articles, the final review included 1 case study, 21 meta-analyses or SLRs, 16 experimental studies, 19 qualitative and 31 quantitative studies, 9 mixed-method studies, and 7 research overviews.

The publications were coded for further information using MAXQDA, a software package for qualitative data analysis, since it is flexible when subcoding is needed.

The codes were developed by the four-member project team according to the realist review (Pawson et al., 2005). The literature review was guided by the question what works for whom, in what circumstances, in what respects, and how. According to the realist review, the focus was on identifying significant contexts, mechanisms, and outcomes of SE programs. The selected literature was finally coded by a single project member. The project team regularly discussed the inclusion and exclusion of literature as well as the codes and their fit. The basic coding included two parts: *Part I* program information (i.e., SE program type, target group, professionals, and other stakeholders); and *Part II* impact factors and impacts separated into context–mechanism–outcome (CMO) regarding RE. This contains the coding to the research question “What components work in supported employment for whom, in what circumstances, in what respect and how?.” We refer to Step 5: Critique the literature—draw conclusions in the following chapter, and to Step 6: Write the review—communicate and evaluate the conclusions in the closing chapter.

## Results

### Type of programs and target groups

*Part I* The analyzed programs were diverse, including clubhouse models (e.g., Torres Stone et al., 2015), cognitive interventions (e.g., Corbière et al., 2013), occupational therapy (e.g., Kirsh et al., 2005), sheltered employment programs (e.g., Verdugo et al., 2006), social skill interventions (e.g., Barreira et al., 2010), and VR programs (e.g., Leahy et al., 2014). Most of the programs assessed were cognitive interventions and VR programs, especially evidence-based SE programs and IPS programs.

For target groups, mainly individuals with disabilities were studied (e.g., Fleming et al., 2013). SE programs, especially IPS programs, are designed for individuals with psychological disability, such as autism spectrum disorder. Additionally, individuals with disabilities included those with developmental disabilities, intellectual disabilities, mental illnesses, brain injuries, or physical disabilities (e.g., Woodall et al., 2017). Other target groups included welfare recipients (e.g., Popp et al., 2017), individuals with HIV or AIDS (Martin et al., 2006), homeless individuals or those abusing substances (e.g., Schutt & Hursh, 2009), and individuals on sick leave or without intellectual disabilities (e.g., Hedley et al., 2017).

Individuals were supervised by vocational agency staff, including rehabilitation counselors, vocational specialists, employment support workers, and job coaches (e.g., Homa & DeLambo, 2015; Williams et al., 2016). Other professionals were case managers, mental health staff, therapists, and interventionists. Some studies mentioned a multidisciplinary team. The main stakeholder groups were employers, the community, or parents, but only a few studies mentioned stakeholder groups that were directly involved in programs (e.g., Woodall et al., 2017).

### Main results: impact factors of SE programs

**Figure 2:**
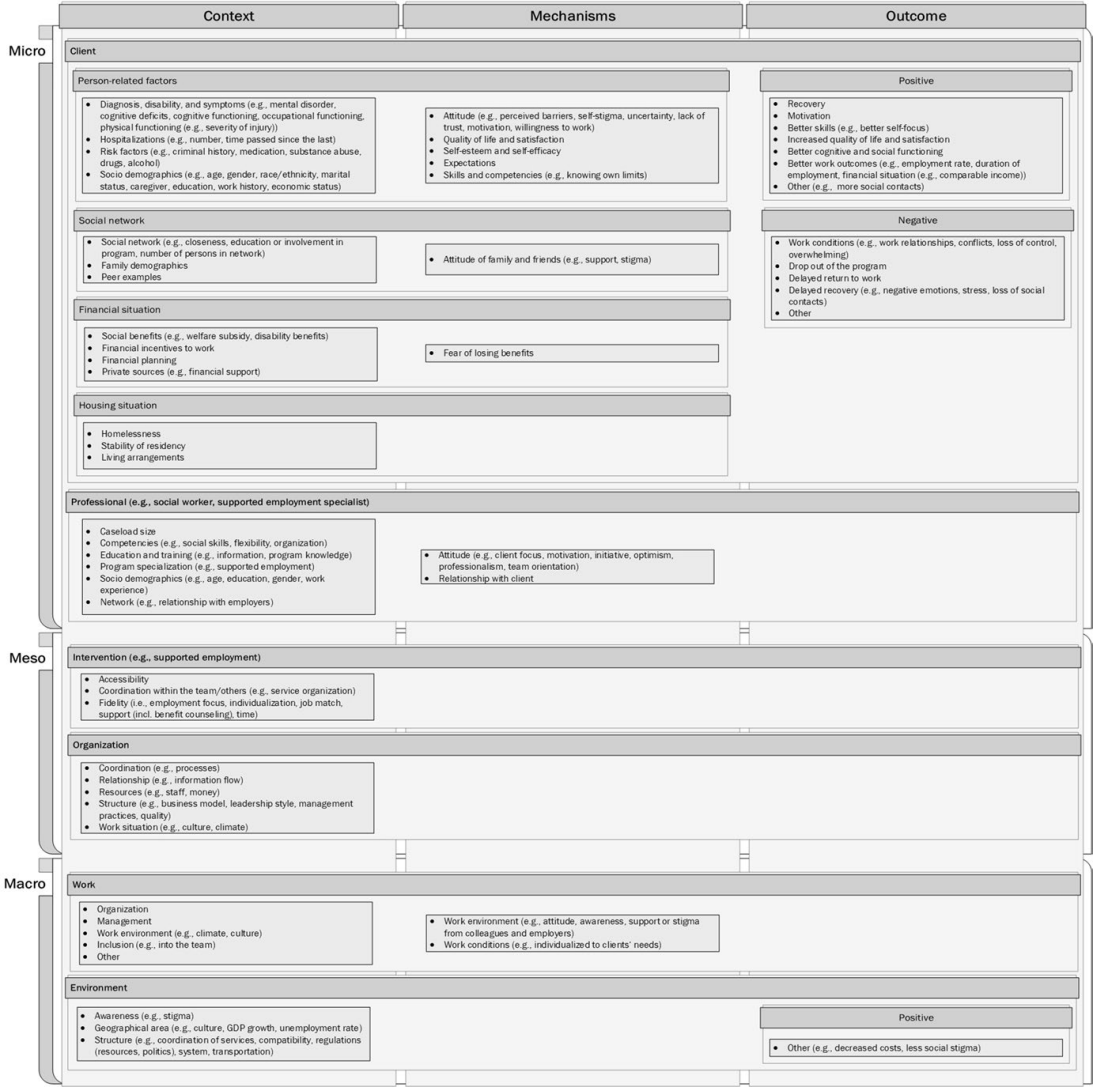
Impact model of supported employment programs

*Part II.* A structure to identify the relevant impact factors, that is, CMO was implemented in MAXQDA. The factors were categorized into three levels. The microsystem level included professionals, clients, their living conditions, financial sources, and networks. The mesosystem level consisted of organizations and interventions. The macrosystem level contained work conditions and environmental factors. Subcoding was used as necessary (see Figure 2).

Outcome is defined as social impact or regularity mechanisms such as composition, behavior, and procedural interaction, which are effective within certain contexts. Context means the framework in which an SE program is established, such as sociodemographics, organizational context, work history, or culture. These are mostly stable over the SE process. Mechanisms describe changes in aspects such as clients’ reasoning (e.g., values, attitudes, etc.) or resources (e.g., information, skills, support, etc.) during the SE program setting. Some contexts might trigger mechanisms while others might not. The interaction between contexts and mechanisms affects the outcomes (or lack thereof) of programs and the CMO hypothesis (Pawson & Tilley, 1997). The resulting impact model clearly illustrates the logic of the consultations.

The results of the SLR strongly suggest that contexts on all levels (microsystem, mesosystem, and macrosystem) shape SE results. The mechanisms are mainly found on the microsystem level, that is, clients and professionals, while some are found on the macrosystem level. The outcomes are mostly related to clients (microsystem level) with the exception of the environment (macrosystem level).

### Impact factors at the microsystem level

The microsystem level of professionals, clients, and their interaction plays an essential role in SE success. The microsystem level contains the main CMO, describing how SE programs work under certain circumstances. An SE program cannot be successful without a participating client (Corbière et al., 2017; Hedley et al., 2017). However, programs work differently; depending on the client’s context, their mechanisms and hence their outcomes might change (Pawson & Tilley, 1997).

The results for contexts are not always unambiguous, especially when it comes to the impact of person-related factors, such as diagnosis, form of disability or symptoms, number of hospitalizations, and risk factors, such as criminal history, medication, substance, drug or alcohol abuse, and sociodemographics. As researchers mentioned, SE studies failed to find consistent results regarding specific client factors (e.g., age, gender, disability status, etc.) affecting the success rates of SE programs (Corbière et al., 2013; Grigorovich et al., 2017). Controversially, other researchers found evidence that these factors do have an influence (Kortrijk et al., 2019). They were supported by other researchers, who found that work experience, illness-related factors, cognitive executive functioning and IQ, as well as other person-related factors affect the outcome of SE programs (Coombes et al., 2016; Hedley et al., 2017).

The same is true for person-related mechanisms, such as attitude, expectations, quality of life and satisfaction, self-esteem and self-efficacy, as well as skills and competencies, which can be shaped during the SE program. Especially, clients’ attitudes contribute to the success of programs. Uncertainty, a lack of trust in one’s own abilities, motivation, willingness to work, self-stigmatization, and perceived barriers also determine success or failure. The more positive the attitude, the more successful the clients (Bond & Drake, 2008; Corbière et al., 2013). Together with others, they highlighted self-esteem, self-efficacy, or internal locus of control as impinging factors to attitude (Bond & Drake, 2008; Corbière et al., 2013). Aligned with this is the impact of perceived quality of life and satisfaction on the success rate of SE programs (Verdugo et al., 2006).

Besides attitude and related factors, expectations, and skills, competencies, and behaviors affect the probability of a successful outcome (Viering et al., 2015; Williams et al., 2016). Social skills, and not the fulfillment of tasks, affect professional success. This was confirmed by Ditchman et al., (2013, p. 352), who found that “[b]ehavior and skills related to general employability.” All these factors affect clients’ ability to work and might be fairly stable but could be influenceable and changeable over time (Fleming et al., 2013).

Regarding the social network of clients, results showed that families and peers influence SE success. For example, clients rated family support as one of the most important factors (Lundqvist & Samuelsson, 2012; Shankar & Collyer, 2014; Torres Stone et al., 2015). Clients need people who acknowledge and motivate them and who are available when support is needed to deal with challenges (Williams et al., 2016). However, one researcher acknowledged that the type of support is essential for success (Corbière et al., 2011).

In connection to this, it is not surprising that social networks act as an important context. The attitude of family and friends was frequently mentioned (e.g., Kirsh, 2016; Lundqvist & Samuelsson, 2012). It was found that families and friends not only help clients find a job with their supportive attitude, but they also contribute to clients’ preservation of these jobs (Hedley et al., 2017). Controversy, stigmatization, or a lack of support by families, partners, and friends functions as a barrier, especially if they show a pessimistic belief about clients’ ability to work (e.g., Corbière et al., 2013; Mueser & McGurk, 2014).

Another interesting context that affects the success of SE programs is the client’s financial situation. Researchers frequently examined the impact of welfare subsidies, disability benefits, or financial support, such as social security disability income and supplemental security income (e.g., Jones et al., 2001). Some researchers mentioned that the results were vague and that financial problems or the financial situation in general, including financial well-being and family financial support, is an obstacle (e.g., Barreira et al., 2010; Fleming et al., 2013; Knaeps et al., 2012). Therefore, the fear of losing benefits, which leads to uncertainty regarding one’s financial situation, is considered as a mechanism in the SE process (Henry & Lucca, 2004). Larson et al., (2007, p. 349) concluded that “participants may have had concern about the impact of employment on government benefits, which may have led to limited incentive to obtain employment.” Thus, researchers recommended that professionals address this fear by using benefit counseling (Gowdy et al., 2004). Another possible solution was identified by Barreira et al., (2010, p. 151), who stated, “[T]he availability of monetary support might help to ameliorate worry about loss of benefits,” considering family financial support. Furthermore, homelessness or housing instability was mentioned as a barrier to finding employment (e.g., Taylor & Bond, 2014).

Altogether, the mentioned contexts and mechanisms determine the outcome of SE programs. Although these are not generally related to the clients, the clients’ positive and negative outcomes were frequently studied. The positive outcomes mentioned are physical or psychological recovery; increased motivation; better skills, such as better self-focus; increased quality of life and satisfaction; better cognitive and social functioning; better work outcomes, such as employment rates, duration of employment, and financial situation; and other outcomes, such as more social contact or normalization of the situation, increased ability to handle stress, and so on (e.g., Hedley et al., 2017; Torres Stone et al., 2015). The negative outcomes examined are mismatched work conditions; higher dropout rates of the program; a delayed return to work because of the SE program; delayed recovery, such as negative effects on psychological and physical health or exhaustion; as well as other outcomes, such as unfulfilled goals, frustration, or social exclusion (e.g., Coombes et al., 2016; Hedley et al., 2017; Verdugo et al., 2006).

Frequently identified contexts of professionals are either organization- or person-related factors. Organization-related factors in particular seem to affect the success of SE programs. Some researchers identified the caseload size as such. However, the results are equivocal, as, for example, some researchers mentioned that larger caseload sizes lead to higher competitive employment rates (e.g., Taylor & Bond, 2014) while others clarified manageable caseloads that can be overseen as one aspect of success. The reasons mentioned are that professionals would have less time to support clients and foster relationships with employers (e.g., Woodall et al., 2017).

Researchers also often mentioned personal factors that might interact, such as competencies, education, and training; specialization; and networking skills. Larson et al. (2014, p. 232) developed a framework that “illuminates employment practitioners’ extensive understanding of knowledge, skills and common factors involved with delivering employment services. Practitioners stressed the need for skilled communicators who bridge the mental health and business environments.” In addition, others identified skills, knowledge, and training as crucial factors (e.g., Coombes et al., 2016; Woodall et al., 2017). Kirsh (2016, p. 814) acknowledged that a “[l]ack of training and knowledge of staff members [...] poses a major barrier.” Some researchers mentioned, besides having good training and knowledge about SE programs (Koletsi et al., 2009), “that the presence of a full-time employment specialist who provided only vocational services was significantly correlated with higher rates of competitive employment” (Kirsh et al., 2005, p. 273), which other researchers also examined (e.g., Viering et al., 2015; Wooff & Schneider, 2006). However, professionals not only need specialized knowledge of SE programs; they must also apply and implement it (Lockett et al., 2018). Knowledge and skills might be connected to some sociodemographic variables since there is evidence that professionals with a master’s degree or longer experience in SE programs record better employment outcomes (e.g., Ditchman et al., 2013; Fleming et al., 2013). Only Viering et al. (2015) found higher success rates if the gender of the professional matches the client’s.

Despite those factors, an additional person-related factor, namely networking skills, is decisive for success. Without networking, which involves building and maintaining relationships with potential employers, the success rate of SE programs decreases rapidly (Corbière et al., 2017; Grigorovich et al., 2017). However, relationship skills also include creating win–win situations for employers and clients (Knaeps et al., 2012) along with obtaining and providing employers with relevant information and communicating adequately with them (Glover & Frounfelker, 2013).

Closely interlinked with those factors are mechanisms. Professionals’ mechanisms are their attitudes and their relationship with clients, which they develop in their interaction with the clients and may change during the process of the intervention (Coombes et al., 2016). Successful professionals show an attitude characterized by client focus, which means that “their focus was to help service users to achieve the outcomes they wanted” (Woodall et al., 2017, p. 48). Professionals who have this focus look beyond the circumstances of the client and work with their strengths and limitations (e.g., Ditchman et al., 2013; Lundqvist & Samuelsson, 2012). Client-focused professionals show an attitude of optimism, hope, enthusiasm, support, patience, acceptance, and flexibility (e.g., Glover & Frounfelker, 2013; Gowdy et al., 2004). As Larson et al., (2014, p. 230) wrote, “[A]n ideal practitioner has a persistent and outgoing personality.” This attitude may be linked to the relationship with a client. Others found that a good working alliance, characterized by a warm, trusting, and respectful colloquial relationship, has a significant impact on the success rates of SE programs (e.g., Gustafsson et al., 2013; Henry & Lucca, 2004; Torres Stone et al., 2015).

### Impact factors at the mesosystem level

At the mesosystem level of successful SE programs, studies have shown contexts regarding interventions and offering organizations. One barrier is access to programs (Viering et al., 2015). As Bond and Drake (2008, p. 364) mentioned, “Lack of access can be explained to some extent by lack of practitioner referrals.” Furthermore, Beimers and Gatlin (2011, p. 5) concluded that “rapid assessment of consumers, rapid approval for employment services, and increased access to services all facilitated entry into employment.”

Another context that might be related to accessibility is the general coordination within the team and with other institutions or specialists (e.g., Fleming et al., 2013; Grigorovich et al., 2017; Knaeps et al., 2012). One study suggested “that higher rates of competitive employment require the active involvement of more than just the supported employment team” (Gowdy et al., 2004, p. 154). Del Valle et al., (2014, pp. 110–111) highlighted the importance of the “exchange between knowledge creation and action to develop and revise services [… to] maximize supports for individuals and increase the opportunities for goal attainment.”

However, coordination within the team can only work if the intervention is properly integrated into the organizational structure. Accordingly, intervention fidelity forms the third identified context that affects the success of SE programs. Fidelity describes prerequisites that an intervention needs to fulfill to produce a reliable outcome. It provides a guideline for organizations to achieve better employment outcomes for their clients (Lockett et al., 2018). Thus, higher fidelity scores are directly linked to the higher success rates of those programs (Bond et al., 2012). In the literature, fidelity is described by seven core principles as mentioned in the introduction (e.g., Beimers & Gatlin, 2011; Bond et al., 2001, 2012; Coombes et al., 2016; Hedley et al., 2017; Knaeps et al., 2012; Larson et al., 2014; Viering et al., 2015).

As can be concluded, different contexts were already revealed in previous studies. Thus, fidelity covers a wider range than just intervention. It also highlights the importance of considering various factors at the same time and indicates that research should not look at interventions in isolation. Therefore, the organizational context plays a role in measuring the impact of SE programs by providing the structures and resources for such programs. It allows the coordination of processes and interventions between teams and other institutions. It promotes relationships and determines the work situation of professionals (Del Valle et al., 2014). McGuire et al. (2011, p. 1067) showed that “vocational services were more effective when integrated with clinical services.” By coordinating knowledge transfer, it can be ensured that clients receive the intervention they need and that all professionals in the organization are informed (Beimers & Gatlin, 2011; Henry & Lucca, 2004).

One aspect of coordination refers to the relationships that are maintained and that influence the flow of information (e.g., Del Valle et al., 2014; Fleming et al., 2013). Research has shown that successful organizations “value relationships with all partners including legislators, employers, community-based rehabilitation organizations, consumer organizations, and the general public” (Leahy et al., 2014, p. 152). Organizations that receive results-based funding, meaning payments only for successful clients, are more effective than organizations with fee-for-service funding (Kirsh, 2016). These findings were supported by other scholars, who also determined the impact of organizational funding on outcomes (e.g., Beimers & Gatlin, 2011). Directly related to funding are resources to train staff and develop innovations (Leahy et al., 2014). Furthermore, researchers showed that the use of advanced information and communication technologies as well as internal case management systems or program evaluations lead to higher success rates by providing best-practice examples and information for all members of the organization (e.g., Bond et al., 2001; Del Valle et al., 2014; Leahy et al., 2014).

However, even if enough resources are available, the structure of the organization and the work situation shape the way in which professionals can work and the success of SE programs. Researchers concluded that the structure, characterized by the business model, leadership style, management practices, and quality criteria (e.g., Larson et al., 2007; Leahy et al., 2014; Wehman et al., 2013), and the culture and climate of an organization (e.g., Beimers & Gatlin, 2011; Del Valle et al., 2014; Fleming et al., 2013; Homa & DeLambo, 2015) are essential in this context.

### Impact factors at the macrosystem level

SE programs work only if there are employers that hire clients for work. Thus, context and mechanism, as well as the type of work for which the clients are hired, influence the success of SE programs. Contexts regarding work are the hiring organization; the organizational management; the work environment, such as the climate, culture, and the client’s inclusion in the team; and others. The hiring organization seems to have an impact, as some researchers found differences concerning the type of organization. For example, Saavedra et al. (2016, p. 855) found that, compared with ordinary companies, “[t]hose who work in social enterprises seem to do so by compensating for important deficits in social (independence-competence) and cognitive functioning (attention, immediate memory and, to a lesser degree, delayed memory).” Furthermore, not only the type of organizations hiring but also their size as well as previous experiences with a client as an employee were also identified as contexts at the macrosystem level (e.g., Grigorovich et al., 2017; Hedley et al., 2017).

Whether an organization hires clients is directly related to management practices (e.g., Hampson et al., 2016; Verdugo et al., 2006). As Hedley et al., (2017, p. 930) stated, “Human resource policies that focus on social skills and teamwork, even when those skills are not essential to the particular workplace or job, create a barrier to entering the workforce for individuals who are perceived as ‘socially awkward.’” There are convictions of the management that function as a barrier to SE programs. Those include concerns about the work capacity of SE program clients and the possibility that they are not comparable to the standards of other employees. Costs related to the employment of SE program clients are a mentioned concern. Management is often “uncertain about the preconditions for and support during the employment” of SE clients (Gustafsson et al., 2013, p. 100). However, if the management is willing to hire SE clients, support from the professional accompanying the client is necessary (Wehman et al., 2013).

After a client is hired, the success of the SE program also depends on the prevailing culture and working atmosphere (Kirsh et al., 2005). A study by Corbière et al., (2013, p. 272) demonstrated “that an organizational culture characterized by discrimination, unfair treatment of workers by supervisors, or job insecurity can determine an individual’s stress reaction that leads them to quit the organization.” These results were supported by other studies, which provided evidence that workplace culture and client inclusion in the team are important in job tenure (Shankar & Collyer, 2014; Williams et al., 2016). The inclusion of clients is determined by the type of job for which they are hired. More typical jobs regarding the rest of the team, which were also mentioned as a context, lead to greater social interaction between colleagues and affect client inclusion (e.g., Verdugo et al., 2006; Wehman et al., 2013; Wooff & Schneider, 2006).

This point is directly related to the mechanisms of the work environment. Work mechanisms include attitudes of employers and colleagues, awareness of the needs of clients, and whether coworkers and employers support or stigmatize clients. As Lundqvist and Samuelsson (2012, p. 1581) emphasized, “having employment and a supporting employer makes it easier to resume a job, which is important for self-image.” It is not surprising that a supportive work environment is frequently mentioned by clients of SE programs (e.g., Bond & Drake, 2008; Kirsh et al., 2009). Moreover, the attitude of the work environment is directly linked to the type of work that clients are offered and whether employers are willing to individualize the job to meet clients’ needs (e.g., Henry & Lucca, 2004; Homa & DeLambo, 2015). Therefore, another mechanism of the work environment includes work conditions (Popp et al., 2017).

As one might conclude, workplace attitude also depends on the environment in which the workplace is located. As Mueser and McGurk (2014, p. S52) stated, “The stigma (and self-stigma) of mental illness is nowhere more apparent than when raising the issue of competitive work for people with serious mental illness.” The awareness that SE programs not only help clients but also bring value to society is a context that allows SE organizations to optimize their programs. Environmental stigma, conversely, is a barrier to SE programs and prevents clients from finding their way back to work (Bond & Drake, 2008; Hampson et al., 2016; Kirsh, 2016).

However, other contexts hamper the success of SE programs. Environmental contexts include the geographical area with its culture, gross domestic product (GDP) growth, and unemployment rates but also the political and societal structures. Researchers determined that unemployment rate and GDP growth might influence the employment rates of SE program clients (e.g., Bond et al., 2012; Fleming et al., 2013; Kirsh, 2016; Viering et al., 2015; Wehman et al., 2013). Other examined contexts included the transportation system (Beimers & Gatlin, 2011; Henry & Lucca, 2004); the childcare system (Henry & Lucca, 2004); the social system and funding of SE programs (Mueser & McGurk, 2014); government policy, health regulations, and insurance policy; and labor laws (Bond & Drake, 2008; Hampson et al., 2016; Kirsh, 2016; Lundqvist & Samuelsson, 2012).

Interestingly, researchers found positive outcomes at the environmental level. They demonstrated the importance of reintegrating SE clients into the labor market. The main outcomes mentioned were decreased costs of the social system and less social stigma. By integrating into the labor market, SE clients cost less, and their reputation increases in the social environment (Bond et al., 2001; Kirsh et al., 2009; Mueser & McGurk, 2014).

Taking all these CMO aspects together, an impact model can be developed (see Figure 2). It reflects the complex interaction of all the factors identified in the underlying SLR. It may also serve as a guide to the practice, pointing to significant factors of SE and providing indicators to the scientific community as to which factors could be further researched.

## Discussion and Conclusion

As noted at the beginning, in contrast to traditional systematic literature reviews, this contribution shows a research synthesis, which is based on the realist approach. It provides an explanatory analysis of SE programs, aimed at discerning what works for whom, in what circumstances, in what respects and how. The review enables the policy and practice community to reach a deeper understanding of SE programs and how it can be made to work most effectively (Pawson et al., 2005).

Although the implementation of VR programs was shown to be effective, practical implementations lag behind theoretical knowledge. Many studies have focused on evidence-based approaches to analyze factors affecting outcomes. Therein, SE was the most frequently used program type, of which the IPS model provided the best outcome for diverse target groups (e.g., Bond et al., 2012; Knaeps et al., 2012). Regarding the research on these SE programs, principles were developed to measure fidelity. Only when high fidelity to the SE model is guaranteed will program implementation lead to effectiveness and good outcomes for individuals (e.g., Bond et al., 2012). Thus, the greatest individual predictor mentioned for positive outcomes is fidelity (Beimers & Gatlin, 2011).

However, besides fidelity and program implementation, there is evidence that other factors shape the effectiveness of SE programs. As Bond et al., (2001, p. 316) stated, “Further research is needed to clarify critical ingredients of supported employment, which will lead to modifications, refinements, and additions.” There were hints at other criteria (i.e., context and mechanism) that affect the outcome of SE programs. Researchers, using a systematic approach, have shown that, besides a good client–employment fit, key variables include individual characteristics as well as contextual factors, cultural and societal attitudes, and environmental factors (Homa & DeLambo, 2015). The fit between clients’ characteristics and the environment, including cultural, social, and physical aspects, is critical. It is important to understand those factors because of their impact on individual behavior and functioning (Homa & DeLambo, 2015). Furthermore, SE corresponds in many ways to ethical social work practice, such as the zero-exclusion criterion or the basic idea of a strength-based approach (Beimers & Gatlin, 2011). Despite that, the relationship between the professional (e.g., the social worker) and the client comes into focus as an influencing factor.

## Suggestions for projecting effective programs

Furthermore, building the model on RE showed that the interaction between contexts and mechanisms affects the outcomes. This interaction generates the results of the program (Pawson & Tilley, 1997). The review showed that most of the research on SE programs were one-dimensional, looking at either the effects of SE programs, the client or the professional, or the relationship between clients and professionals. The model reveals that impact factors are interconnected (e.g., if employers and the work environment are open to creating working conditions that suit the client’s needs, the satisfaction with these conditions increases and accordingly the possibility of lengthening work tenure). There are cumulative impacts on the client, the professional, the work environment, and so on. By considering more impact factors in the coproduction process with the client, professionals can offer even more individualized SE programs, respond quickly to changing circumstances, and measure the impact of their intervention. This might help in dealing with barriers that otherwise become too challenging and outweigh the support. To plan effective programs, we particularly recommend facilitating organizational change and trusting team relationships (especially the integration of mental health and vocational services within a single service team), clarifying and discussing anticipated gains of integration, and considering continuous support for sustainability (Hillborg et al., 2020).

## Suggestions for further studies

Thus, this review provides the groundwork for further studies to make SE programs even more effective by building on CMO configurations at the microsystem, mesosystem, and macrosystem levels. A special focus still needs to be on the microsystem level, meaning the relationship between clients and professionals, especially, as actions at the microsystem level are integrated into higher levels. More research is needed to understand how social workers and therapists experience their contribution to IPS (Moen et al., 2020) and to understand better ongoing support and attention to client preferences as well as the correlation between those with SMI and employment outcomes (Bender et al., 2020).

Even though this study provides an expanded impact model for considering SE programs, the limitations of the work must also be presented. First, only German and English publications and only four databases were considered. Notwithstanding the presentation of an expansive SLR, there might still be unlocated articles that meet the inclusion criteria and are relevant to completing the impact model developed. Second, the focus was only on identifying impact factors with some initial evidence of effectiveness; therefore, no quality criteria for the studies were collected and evaluated. Third, certain VR programs were selected based on the selection criteria, which could also contribute to further studies.

## Supplementary Information

Below is the link to the electronic supplementary material.

Supplementary file1 (DOCX 37 kb)

## Footnotes

1 The systematic literature review is part of a Swiss National Science Foundation (SNSF)-funded research project on impact factors in social organizations.

2 Because the aim of the research was to review the German-language evidence, but also to specifically consider the more advanced international state of research, keywords in both languages were used.
