# Supplementary material for: How do supported employment programs work? Answers from a systematic literature review
Source: Int J Educ Vocat Guid. 2022 Feb 27;23(3):659–79. doi: 10.1007/s10775-022-09533-3 (PMC10444632; doi:10.1007/s10775-022-09533-3)
Supplement: Supplementary file 1 — Supplementary file1 (DOCX 37 kb) [file 10775_2022_9533_MOESM1_ESM.docx]

# Appendix A – Systematic Literature Review – Articles

1. Baker-Ericzén, M. J., Fitch, M. A., Kinnear, M., Jenkins, M. M., Twamley, E. W., Smith, L., Montano, G., Feder, J., Crooke, P. J., Winner, M. G., & Leon, J. (2018). Development of the supported employment, comprehensive cognitive enhancement, and social skills program for adults on the autism spectrum: Results of initial study. *Autism*, 22 (1), 6–19. https://doi.org/10.1177/1362361317724294.
2. Baksheev, G. N., Allott, K., Jackson, H. J., McGorry, P. D., & Killackey, E. (2012). Predictors of Vocational recovery among young people with first-episode psychosis: Findings from a randomized controlled trial. *Psychiatric Rehabilitation Journal,* 35 (6), 421–427. https://doi.org/10.1037/h0094574.
3. Barreira, P. J., Tepper, M. C., Gold, P. B., Holley, D., & Macias, C. (2010). Adapting evidence-based interventions to fit usual practice: Staff roles and consumer choice in psychiatric rehabilitation. Psychiatric Quarterly, 81(2), 139–155. https://doi.org/10.1007/s11126-010-9124-4.
4. Beimers, D., & Gatlin, E. (2011). Supported employment in rural areas: Implications for mental health practice. Journal of Rural Mental Health, 35(2), 3–11. https://doi.org/10.1037/h0094769.
5. Biegel, D. E., Beimers, D., Stevenson, L. D., Ronis, R. J., & Boyle, P. (2009). Predictors of Referral to supported employment among consumers with co-occurring mental and substance use disorders. *Community Mental Health Journal*, 45 (6), 427–438. https://doi.org/10.1007/s10597-009-9242-3.
6. Bond, G. R. (2004). Supported employment: Evidence for an evidence-based practice. *Psychiatric Rehabilitation Journal*, 27 (4), 345–359. https://doi.org/10.2975/27.2004.345.359.
7. Bond, G. R., & Drake, R. E. (2008). Predictors of competitive employment among patients with schizophrenia. Current Opinion in Psychiatry, 21(4), 362–369. https://doi.org/ 10.1097/YCO.0b013e328300eb0e.
8. Bond, G. R., Becker, D. R., Drake, R. E., Rapp, C. A., Meisler, N., Lehman, A. F., Bell, M. D., & Blyler, C. R. (2001). Implementing supported employment as an evidence-based practice. Psychiatric Services, 52(3), 313–322. https://doi.org/10.1176/appi.ps.52.3.313.
9. Bond, G. R., Peterson, A. E., Becker, D. R., & Drake, R. E. (2012). Validation of the revised individual placement and support fidelity scale (IPS-25). Psychiatric Services, 63(8), 758–763. https://doi.org/10.1176/appi.ps.201100476.
10. Bowie, C. R., Grossman, M., Gupta, M., Holshausen, K., & Best, M. W. (2017). Action-based cognitive remediation for individuals with serious mental illnesses: Effects of real-world simulations and goal setting on functional and vocational outcomes. *Psychiatric Rehabilitation Journal*, 40 (1), 53–60. https://doi.org/10.1037/prj0000189.
11. Brantschen, E., Landolt, K., Kawohl, W., Rössler, W., Bärtsch, B., & Nordt, C. (2017). Two Types of expectancies concerning competitive employment among people with mental illness in supported employment. *Journal of Vocational Rehabilitation*, 46 (2), 195–202. https://doi.org/10.3233/JVR-160855.
12. Burns, T., Catty, J., Becker, T., Drake, R.E., Fioritti, A., Knapp, M., Lauber, C., Rossler, W., Tomov, T., van, B.J., White, S., & Wiersma, D. (2007). The effectiveness of supported employment for people with severe mental illness: A randomised controlled trial. *The Lancet*, 370 (9593), 1146–1152. https://doi.org/10.1016/S0140-6736(07)61516-5.
13. Catalano, D., Pereira, A. P., Wu, M., Ho, H., & Chan, F. (2006). Service patterns related to successful employment outcomes of persons with traumatic brain injury in vocational rehabilitation. *NeuroRehabilitation*, 21 (4), 279–293.
14. Catty, J., Lissouba, P., White, A., Becker, T., Drake, R. E., Fioritti, A., Knapp, M., Lauber, C., Rössler, W., Tomov, T., van Busschbach, J., Wiersma, D., Burns, T., & EQOLISE Group (2008). Predictors of employment for people with severe mental illness: Results of an international six-centre randomised controlled trial. *The British Journal of Psychiatry*, 192 (3), 224–231. https://doi.org/10.1192/bjp.bp.107.041475.
15. Cook, J. A., & Razzano, L. (2000). Vocational Rehabilitation for persons with schizophrenia: recent research and implications for practice. *Schizophrenia Bulletin*, 26 (1), 87–103. https://doi.org/10.1093/oxfordjournals.schbul.a033448.
16. Cook, J. A., Burke-Miller, J. K., & Roessel, E. (2016). Long-term effects of evidence-based supported employment on earnings and on ssi and ssdi participation among individuals with psychiatric disabilities. *American Journal of Psychiatry*, 173 (10), 1007–1014. https://doi.org/10.1176/appi.ajp.2016.15101359.
17. Cook, J. A., Lehman, A. F., Drake, R., McFarlane, W. R., Gold, P. B., Leff, H. S., Blyler, C., Toprac, M. G., Razzano, L. A., Burke-Miller, J. K., Blankertz, L., Shafer, M., Pickett-Schenk, S. A., & Grey, D. D. (2005). Integration of psychiatric and vocational services: A multisite randomized, controlled trial of supported employment. *American Journal of Psychiatry*, 162 (10), 1948–1956. https://doi.org/10.1176/appi.ajp.162.10.1948.
18. Coombes, K., Haracz, K., Robson, E., James, C. (2016). Pushing through: Mental health consumers’ experiences of an individual placement and support employment programme. British Journal of Occupational Therapy, 79(11), 651–659. https://doi.org/10.1177/0308022616658297.
19. Corbière, M., Lecomte, T., Reinharz, D., Kirsh, B., Goering, P., Menear, M., Berbiche, D., Genest, K., & Goldner, E. M. (2017). Predictors of acquisition of competitive employment for people enrolled in supported employment programs. Journal of Nervous and Mental Disease, 205(4), 275–282. https://doi.org/10.1097/NMD.0000000000000612.
20. Corbière, M., Negrini, A., & Dewa, C. S. (2013). Mental health problems and mental disorders: Linked determinants to work participation and work functioning. In Loisel, P., & Anema, J. R. (Eds). Handbook of work disability: Prevention and management (pp. 267–288). New York, NY: Springer. https://doi.org/10.1007/978-1-4614-6214-9_17.
21. Corbière, M., Zaniboni, S., Lecomte, T., Bond, G., Gilles, P. Y., Lesage, A., & Goldner, E. (2011). Job acquisition for people with severe mental illness enrolled in supported employment programs: A theoretically grounded empirical study. *Journal of Occupational Rehabilitation,* 21 (3), 342–354. https://doi.org/10.1007/s10926-011-9315-3.
22. De Urríes, F. B. J., Verdugo, M. A., Jenaro, C., Crespo, M., & Caballo, C. (2005). Supported employment and job outcomes. Typicalness and other related variables. *Work*, 25 (3), 221–229.
23. Del Valle, R., Leahy, M. J., Sherman, S., Anderson, C. A., Tansey, T., & Schoen, B. (2014). Promising best practices that lead to employment in vocational rehabilitation: Findings from a four-state multiple case study. Journal of Vocational Rehabilitation, 41(2), 99–113. https://doi.org/10.3233/JVR-140708.
24. Dewa, C. S., Loong, D., Trojanowski, L., & Bonato, S. (2018). The effectiveness of augmented versus standard individual placement and support programs in terms of employment: A systematic literature review. *Journal of Mental Health*, 27 (2), 174–183. https://doi.org/10.1080/09638237.2017.1322180.
25. Ditchman, N., Wu, M.-Y., Chan, F., Fitzgerald, S., Lin, C., & Tu, W. (2013). Vocational rehabilitation. In Strauser, D. R. (Ed). Career development, employment, and disability in rehabilitation: From theory to practice (pp. 343–360). New York, NY: Springer Publishing Company.
26. Donker-Cools, B. H. P. M., Daams, J. G., Wind, H., & Frings-Dresen, M. H. (2016). Effective return-to-work interventions after acquired brain injury: A systematic review. *Brain Injury*, 30 (2), 113–131. https://doi.org/10.3109/02699052.2015.1090014.
27. Drake, R. E., Bond, G. R., & Rapp, C. (2006). Explaining the variance within supported employment programs: Comment on ‘what predicts supported employment outcomes?’ *Community Mental Health Journal*, 42 (3), 315–318. https://doi.org/10.1007/s10597-006-9038-7.
28. Fadyl, J. K., & McPherson, K. M. (2009). Approaches to vocational rehabilitation after traumatic brain injury: A review of the evidence. *The Journal of Head Trauma Rehabilitation*, 24 (3), 195-212. https://doi.org/10.1097/HTR.0b013e3181a0d458.
29. Falkum, E., Klungsøyr, O., Lystad, J. U., Bull, H. C., Evensen, S., Martinsen, E. W., Friis, S., & Ueland, T. (2017). Vocational rehabilitation for adults with psychotic disorders in a scandinavian welfare society. *BMC Psychiatry*, 17 (1), 24–34. https://doi.org/10.1186/s12888-016-1183-0.
30. Ferguson, K. M., Xie, B., & Glynn, S. (2012). Adapting the Individual placement and support model with homeless young adults. *Child & Youth Care Forum*, 41 (3), 277–294. https://doi.org/10.1007/s10566-011-9163-5.
31. Fleming, A. R., Del Valle, R., Kim, M., & Leahy, M. J.(2013). Best practice models of effective vocational rehabilitation service delivery in the public rehabilitation program: A review and synthesis of the empirical literature. Rehabilitation Counseling Bulletin, 56(3), 146–159. https://doi.org/10.1177/0034355212459661.
32. Glover, C. M., & Frounfelker, R. L. (2013). Competencies of more and less successful employment specialists. Community Mental Health Journal, 49(3), 311–316. https://doi.org/10.1007/s10597-011-9471-0.
33. Gowdy, E. A., Carlson, L. S., & Rapp, C. A. (2004). Organizational factors differentiating high performing from low performing supported employment programs. Psychiatric Rehabilitation Journal, 28(2), 150–156. https://doi.org/10.2975/28.2004.150.156.
34. Grigorovich, A., Stergiou-Kita, M., Damianakis, T., Le Dorze, G., Lemsky, C., & Hebert, D. (2017). Persons with brain injury and employment supports: Long-term employment outcomes and use of community-based services. Brain Injury, 31(5), 607–619. https://doi.org/10.1080/02699052.2017.1280855.
35. Gustafsson, J., Peralta, J. P., & Danermark, B. (2013). The employer’s perspective on supported employment for people with disabilities: Successful approaches of supported employment organizations. Journal of Vocational Rehabilitation, 38(2), 99–111. https://doi.org/10.3233/JVR-130624.
36. Hampson, M., Hicks, R., & Watt, B. (2016). Understanding the employment barriers and support needs of people living with psychosis. The Qualitative Report, 21(5), 870–886.
37. Hedley, D., Uljarević, M., Cameron, L., Halder, S., Richdale, A., & Dissanayake, C. (2017). Employment programmes and interventions targeting adults with autism spectrum disorder: A systematic review of the literature. Autism, 21(8), 929–941. https://doi.org/10.1177/1362361316661855.
38. Henry, A. D., & Lucca, A. M. (2004). Facilitators and barriers to employment: The perspectives of people with psychiatric disabilities and employment service providers. Work, 22(3), 169–182.
39. Hillborg, H., Danermark, B., & Svensson, T. (2013). Professionals’ perceptions of and views about vocational rehabilitation for people with psychiatric disabilities. *Work*, 44 (4), 471–480. https://doi.org/10.3233/WOR-131518.
40. Hoffmann, H., Kupper, Z., & Kunz, B. (2001). Vocational rehabilitation in schizophrenia — New findings in outcome prediction. In: Brenner HD and Boker W (ed.) *The treatment of schizophrenia, status and emerging trends.* Seattle, WA: Hogrefer & Huber, pp*.* 225–246.
41. Homa, D., & DeLambo, D. (2015). Vocational assessment and job placement. In Escorpizo, R., Brage, S., Homa, D., Stucki, G. (Eds). Handbook of vocational rehabilitation and disability evaluation: Application and implementation of the ICF (pp. 161–186). Handbooks in health, work, and disability. Cham: Springer International Publishing. https://doi.org/10.1007/978-3-319-08825-9_8.
42. Jang, Y., Wang, Y., & Lin, M. (2014). Factors affecting employment outcomes for people with disabilities who received disability employment services in Taiwan. *Journal of Occupational Rehabilitation*, 24 (1), 11–21. https://doi.org/10.1007/s10926-013-9433-1.
43. Jones, C. J., Perkins, D. V., & Born, D. L. (2001). Predicting work outcomes and service use in supported employment services for persons with psychiatric disabilities. Psychiatric Rehabilitation Journal, 25(1), 53–59. https://doi.org/10.1037/h0095050.
44. Kirsh, B. (2016). Client, contextual and program elements influencing supported employment: A literature review. Community Mental Health Journal, 52(7), 809–820. https://doi.org/10.1007/s10597-015-9936-7.
45. Kirsh, B., Cockburn, L., & Gewurtz, R. (2005). Best practice in occupational therapy: Program characteristics that influence vocational outcomes for people with serious mental illnesses. Canadian Journal of Occupational Therapy, 72(5), 265–279. https://doi.org/10.1177/000841740507200503.
46. Kirsh, B., Stergiou-Kita, M., Gewurtz, R., Dawson, De., Krupa, T., Lysaght, R., & Shaw, L. (2009). From margins to mainstream: What do we know about work integration for persons with brain injury, mental illness and intellectual disability? Work, 32(4), 391–405. https://doi.org/10.3233/WOR-2009-0851.
47. Knaeps, J., DeSmet, A., & Van Audenhove, C. (2012). The IPS fidelity scale as a guideline to implement supported employment. Journal of Vocational Rehabilitation, 37(1), 13–23. https://doi.org/10.3233/JVR-2012-0596.
48. Koletsi, M., Niersman, A., van Busschbach, J. T., Catty, J., Becker, T., Burns, T., Fioritti, A., Kalkan, R., Lauber, C., Rössler, W., Tomov, T., & Wiersma, D. (2009). Working with mental health problems: Clients’ experiences of IPS, vocational rehabilitation and employment. Social Psychiatry and Psychiatric Epidemiology, 44(11), 961–970. https://doi.org/10.1007/s00127-009-0017-5.
49. Kortrijk, H. E., Mulder, N. L., Kamperman, A. M., & van Weeghel, J. (2019). Employment rates in flexible assertive community treatment teams in the Netherlands: An observational study. Community Mental Health Journal, 55(2), 350–359. https://doi.org/10.1007/s10597-018-0233-0.
50. Kukla, M., Strasburger, A. M., Salyers, M. P., Rattray, N. A., & Lysaker, P. H. (2017). Subjective experiences of the benefits and key elements of a cognitive behavioral intervention focused on community work outcomes in persons with mental illness. *The Journal of Nervous and Mental Disease*, 205 (1), 66–73. https://doi.org/10.1097/NMD.0000000000000601.
51. Larson, J. E., Barr, L. K., Kuwabara, S. A., Boyle, M. G., & Glenn, T. L. (2007). Process and outcome analysis of a supported employment program for people with psychiatric disabilities. American Journal of Psychiatric Rehabilitation, 10(4), 339–353. https://doi.org/10.1080/15487760701680604.
52. Larson, J. E., Sheehan, L., Ryan, C., Lemp, S., & Drandorff, L. (2014). Practitioner perspectives on individual placement and support (IPS) for individuals with serious mental illness. Journal of Vocational Rehabilitation, 41(3), 225–235. https://doi.org/10.3233/JVR-140715.
53. Leahy, M. J., Chan, F., Lui, J., Rosenthal, D., Tansey, T., Wehman, P., Kundu, M., Dutta, A., Anderson, C. A., Del Valle, R., Sherman, S., & Menz, F. E. (2014). An analysis of evidence-based best practices in the public vocational rehabilitation program: Gaps, future directions, and recommended steps to move forward. Journal of Vocational Rehabilitation, 41(2), 147–163. https://doi.org/10.3233/JVR-140707.
54. Leddy, M., Stefanovics, E., & Rosenheck, R. (2014). Health and well-being of homeless veterans participating in transitional and supported employment: Six-month outcomes. *Journal of Rehabilitation Research & Development*, 51 (1), 161–174. https://doi.org/10.1682/JRRD.2013.01.0011.
55. LePage, J. P., Bluitt, M., House-Hatfield, T., McAdams, H., Burdick, M., Dudley, D., Michaels, S., Merrell, C., Otto, S., Lenger-Gvist, J., Eisworth, J., Newton, J. A., & Gaston, C. (2005). Improving success in a veterans homeless domiciliary vocational program: Model development and Evaluation. *Rehabilitation Psychology*, 50 (3), 297–304. https://doi.org/10.1037/0090-5550.50.3.297.
56. Lexén, A., Hofgren, C., & Bejerholm, U. (2013). Support and process in individual placement and support: A multiple case study. *Work*, 44 (4), 435–448. https://doi.org/10.3233/WOR-2012-1360.
57. Lockett, H., Waghorn, G., & Kydd, R. (2018). A framework for improving the effectiveness of evidence-based practices in vocational rehabilitation. Journal of Vocational Rehabilitation, 49(1), 15–31. https://doi.org/0.3233/JVR-180951.
58. Lundqvist, A., & Samuelsson, K. (2012). Return to work after acquired brain injury: A patient perspective. Brain Injury, 26(13–14), 1574–1585. https://doi.org/10.3109/02699052.2012.698363.
59. Macias, C., Jones, D. R., Hargreaves, W. A., Wang, Q., Rodican, C. F., Barreira, P. J., & Gold, P. B. (2008). When programs benefit some people more than others: Tests of differential service effectiveness. *Administration and Policy in Mental Health and Mental Health Services Research*, 35 (4), 283–294. https://doi.org/10.1007/s10488-008-0174-y.
60. Martin, D. J., Arns, P. G., Batterham, P. J., Afifi, A. A., & Steckart, M. J. (2006). Workforce reentry for people with HIV/AIDS: Intervention effects and predictors of success. Work, 273, 221–233.
61. McGuire, A. B., Bond, G. R., Clendenning, D. R., & Kukla, M. (2011). Service intensity as a predictor of competitive employment in an individual placement and support model. Psychiatric Services, 62(9), 1066–1072. https://doi.org/10.1176/ps.62.9.pss6209_1066
62. McGurk, S. R., & Mueser, K. T. (2004). Cognitive Functioning, symptoms, and work in supported employment: A review and heuristic model. *Schizophrenia Research*, 70 (2), 147–173. https://doi.org/10.1016/j.schres.2004.01.009.
63. McGurk, S. R., & Mueser, K. T. (2006). Strategies for coping with cognitive impairments of clients in supported employment. *Psychiatric Services,* 57 (10), 1421–1429. https://doi.org/10.1176/ps.2006.57.10.1421.
64. McGurk, S. R., Mueser, K. T., & Pascaris, A. (2005.) Cognitive training and supported employment for persons with severe mental illness: One-year results from a randomized controlled trial. *Schizophrenia Bulletin,* 31 (4), 898–909. https://doi.org/10.1093/schbul/sbi037.
65. McGurk, S. R., Mueser, K. T., Feldman, K., Wolfe, R., & Pascaris, A. (2007). Cognitive training for supported employment: 2-3 year outcomes of a randomized controlled trial. *American Journal of Psychiatry*, 164 (3), 437–441.
66. Michon, H. W. C., van Weeghel, J., Kroon, H., & Schene, A. H. (2005). Person-related predictors of employment outcomes after participation in psychiatric vocational rehabilitation programmes. *Social Psychiatry and PsychiatricEpidemiology,* 40 (5), 408–416. https://doi.org/10.1007/s00127-005-0910-5.
67. Michon, H. W. C., van Weeghel, J., Kroon, H., Smit, F., & Schene, A. H. (2006). Predictors of successful job finding in psychiatric vocational rehabilitation: An expert panel study. *Journal of Vocational Rehabilitation*, 25 (3), 161–171.
68. Migliore, A., Butterworth, J., Nord, D., Cox, M., & Gelb, A. (2012), Implementation of job development practices. *Intellectual and Developmental Disabilities,* 50 (3), 207–218. https://doi.org/10.1352/1934-9556-50.3.207.
69. Modini, M., Tan, L., Brinchmann, B., Wang, M., Killackey, E., Glozier, N., Mykletun, A., & Harvey, S. B. (2016). Supported employment for people with severe mental illness: Systematic review and meta-analysis of the international evidence. *The British Journal of Psychiatry,* 209 (1), 14–22. https://doi.org/10.1192/bjp.bp.115.165092.
70. Mueser, K. T., & McGurk, S. R. (2014). Supported employment for persons with serious mental illness: Current status and future directions. L’Encéphale, 40(2). Insertion professionnelle et handicap psychique, S45–S56. https://doi.org/10.1016/j.encep.2014.04.008.
71. Mueser, K. T., Aalto, S., Becker, D. R., Ogden, J. S., Wolfe, R.S., Schiavo, D., Wallace, C. J., & Xie, H. (2005). The effectiveness of skills training for improving outcomes in supported employment. *Psychiatric Services*, 56 (10), 1254–1260. https://doi.org/10.1176/appi.ps.56.10.1254.
72. Mueser, K. T., Bond, G. R., Essock, S. M., Clark, R. E., Carpenter-Song, E., Drake, R. E., & Wolfe, R. (2014). The Effects of supported employment in latino consumers with severe mental illness. *Psychiatric Rehabilitation Journal,* 37 (2), 113–122. https://doi.org/10.1037/prj0000062.
73. Muñoz-Murillo, A., Esteban, E., Ávila, C. C., Fheodoroff, K., Haro, J. M., Leonardi, M., & Olaya, B. (2018). Furthering the evidence of the effectiveness of employment strategies for people with mental disorders in Europe: A systematic review. *International Journal of Environmental Research and Public Health*, 15 (5), 838–856. https://doi.org/10.3390/ijerph15050838.
74. Nygren, U., Markström, U., Bernspång, B., Svensson, B., Hansson, L., & Sandlund, M. (2013). Predictors of vocational outcomes using individual placement and support for people with mental illness. *Work*, 45 (1), 31–39. https://doi.org/10.3233/WOR-131598.
75. Perkins, D. (2008). Improving employment participation for welfare recipients facing personal barriers. *Social Policy and Society*, 7 (1), 13–26. https://doi.org/10.1017/S1474746407003971.
76. Phillips, B. N., Kaseroff, A. A., Fleming, A. R., Huck, G. E. (2014). Work-related social skills: Definitions and interventions in public vocational rehabilitation. *Rehabilitation Psychology*, 59 (4), 386–398. https://doi.org/10.1037/rep0000011.
77. Pittam, G., Boyce, M., Secker, J., Lockett, H., & Samele, C. (2010). Employment advice in primary care: A realistic evaluation. *Health & Social Care in the Community*, 18 (6), 598–606. https://doi.org/10.1111/j.1365-2524.2010.00929.x.
78. Popp, S., Kupka, P., Gühne, U., Riedel-Heller, S., & Oschmiansky, F. (2017). Psychisch kranke Leistungsberechtigte im SGB II: Barrieren und Gelingensbedingungen bei der Integration in den Arbeitsmarkt. ARCHIV für Wissenschaft und Praxis der sozialen Arbeit (4/2017). Vierteljahresschrift zur Förderung von Sozial-, Jugend- und Gesundheitshilfe, 52–61.
79. Puig, O., Thomas, K. R., & Twamley, E. W. (2016). Age and improved attention predict work attainment in combined compensatory cognitive training and supported employment for people with severe mental illness. *The Journal of Nervous and Mental Disease*, 204 (11), 869–872. https://doi.org/10.1097/NMD.0000000000000604.
80. Rashid, M., Hodgetts, S., & Nicholas, D. (2017). Building employer capacity to support meaningful employment for persons with developmental disabilities: A grounded theory study of employment support perspectives. *Journal of Autism and Developmental Disorders*, 47 (11), 3510–3519. https://doi.org/10.1007/s10803-017-3267-1.
81. Razzano, L. A., Cook, J. A., Burke-Miller, J. K., Mueser, K. T., Pickett-Schenk, S. A., Grey, D. D., Goldberg, R. W., Blyler, C. R., Gold, P. B., Leff, H. S., Lehman, A. F., Shafer, M. S., Blankertz, L. E., McFarlane, W. R., Toprac, M. G., & Ann Carey, M. (2005). Clinical factors associated with employment among people with severe mental illness: findings from the employment intervention demonstration program. *The Journal of Nervous and Mental Disease*, 193 (11), 705–XX. https://doi.org/10.1097/01.nmd.0000185939.11282.3e.
82. Resnick, S. G., Rosenheck, R. A., & Drebing, C. E. (2006). what makes vocational rehabilitation effective? Program characteristics versus employment outcomes nationally in VA. *Psychological Services*, 3 (4), 239–248. https://doi.org/10.1037/1541-1559.3.4.239.
83. Rosenthal, D. A., Dalton, J. A., & Gervey, R. (2007). Analyzing vocational outcomes of individuals with psychiatric disabilities who received state vocational rehabilitation services: A data mining approach. *International Journal of Social Psychiatry*, 53 (4), 357–368. https://doi.org/10.1177/0020764006074555.
84. Saavedra, J., López, M., González, S., Arias, S., & Crawford, P. (2016). Cognitive and social functioning correlates of employment among people with severe mental illness. Community Mental Health Journal, 52(7), 851–858. https://doi.org/10.1007/s10597-015-9874-4.
85. Schindler, V. P., & Kientz, M. (2013). Supports and barriers to higher education and employment for individuals diagnosed with mental illness. *Journal of Vocational Rehabilitation*, 39 (1), 29–41. https://doi.org/10.3233/JVR-130640.
86. Schutt, R. K., & Hursh, N. C. (2009). Influences on job retention among homeless persons with substance abuse or psychiatric disabilities. Journal of Sociology and Social Welfare, 36(4), 53–73.
87. Shankar, J., & Collyer, F. (2014). Vocational rehabilitation of people with mental illness: The need for a broader approach. Australian e-Journal for the Advancement of Mental Health, 2(2), 77–89. https://doi.org/10.5172/jamh.2.2.77.
88. Storey, K. (2002). Strategies for increasing interactions in supported employment settings: An updated review. *Journal of Vocational Rehabilitation*, 17 (4), 231–237.
89. Tan, B., Li, Z., & Tan, C. (2016). Evaluation of a national supported employment programme for people with psychiatric conditions. *British Journal of Occupational Therapy*, 79 (5), 270–274. https://doi.org/10.1177/0308022615615891.
90. Taylor, A. C., & Bond, G. R. (2014). Employment specialist competencies as predictors of employment outcomes. Community Mental Health Journal, 50(1), 31–40. https://doi.org/10.1007/s10597-012-9554-6.
91. Torres Stone, R. A., Delman, J., McKay, C. E., & Smith, L. M. (2015). Appealing features of vocational support services for Hispanic and non-Hispanic transition age youth and young adults with serious mental health conditions. Journal of Behavioral Health Services & Research, 42(4), 452–465. https://doi.org/10.1007/s11414-014-9402-2.
92. Tsang, H. W. H., Chan, A., Wong, A., & Liberman, R. P. (2009). Vocational outcomes of an integrated supported employment program for individuals with persistent and severe mental illness. *Journal of Behavior Therapy and Experimental Psychiatry,* 40 (2), 292–305. https://doi.org/10.1016/j.jbtep.2008.12.007.
93. Tsang, H. W. H., Leung, A. Y., Chung, R. C. K., Bell, M., & Cheung, W. M. (2010). Review on vocational predictors: A systematic review of predictors of vocational outcomes among individuals with schizophrenia: An update since 1998. *Australian and New Zealand Journal of Psychiatry*, 44 (6), 495–504.
94. van Velzen, J. M., van Bennekom, C. A. M., van Dormolen, M., Sluiter, J. K., & Frings-Dresen, M. H. (2016). Evaluation of the implementation of the protocol of an early vocational rehabilitation intervention for people with acquired brain injury. *Disability and Rehabilitation*, 38 (1), 62–70. https://doi.org/10.3109/09638288.2015.1017057.
95. Verdugo, M. A., Jordán de Urríes, F. B., Jenaro, C., Caballo, C., & Crespo, M. (2006). Quality of life of workers with an intellectual disability in supported employment. Journal of Applied Research in Intellectual Disabilities, 19(4), 309–316. https://doi.org/10.1111/j.1468-3148.2006.00277.x.
96. Viering, S., Jäger, M., & Kawohl, W. (2015). Welche Faktoren beeinflussen den Erfolg von Supported Employment? Psychiatrische Praxis, 42(6), 299–308. https://doi.org/10.1055/s-0034-1387695.
97. Watzke, S., Galvao, A., Gawlik, B., Huehne, M., & Brieger, P. (2006). Change in work performance in vocational rehabilitation for people with severe mental illness: Distinct responder groups. *International Journal of Social Psychiatry,* 52 (4), 309–323. https://doi.org/10.1177/0020764006065141.
98. Waynor, W. R., & Pratt, C. W. (2011). Barriers to vocational effectiveness in ACT: Staff perspectives. *Journal of the American Psychiatric Nurses Association,* 17 (1), 72–79. https://doi.org/10.1177/1078390310394844.
99. Wehman, P. H., Targett, P. S., & West, M. D. (2013). Supported employment/customized employment. In Strauser, D. R. (Ed.), Career development, employment, and disability in rehabilitation: From theory to practice (pp. 325–341). New York, NY: Springer Publishing Company.
100. Williams, A. E., Fossey, E., Corbière, M., Paluch, T., & Harvey, C. (2016). Work participation for people with severe mental illnesses: An integrative review of factors impacting job tenure. Australian Occupational Therapy Journal, 63(2), 65–85. https://doi.org/10.1111/1440-1630.12237.
101. Wisenthal. A., Krupa, T., Kirsh, B. H., & Lysaght, R. (2018). Cognitive work hardening for return to work following depression: An intervention study. *Canadian Journal of Occupational Therapy*, 85 (1), 21–32. https://doi.org/10.1177/0008417417733275.
102. Woodall, J., Southby, K., Trigwell, J., Lendzionowski, V., & Rategh, R. (2017). Maintaining employment and improving health. International Journal of Workplace Health Management, 10(1), 42–54. http://dx.doi.org/10.1108/IJWHM-02-2016-0005.
103. Wooff, D. A., & Schneider, J. M. (2006). A Bayesian belief network for quality assessment: Application to employment officer support. Journal of Intellectual Disability Research, 50(2), 109–126. https://doi.org/10.1111/j.1365-2788.2005.00736.x.
104. Yun-Tung, W. (2010). Job coach factors associated with community-based employment service programme outcome measures for people with disabilities – a Taiwan case study. *Disability and Rehabilitation* ,32 (19), 1547–1557. https://doi.org/10.3109/09638281003599604.
